# Raman Spectroscopic Characterizations of Self-Catalyzed InP/InAs/InP One-Dimensional Nanostructures on InP(111)B Substrate using a Simple Substrate-Tilting Method

**DOI:** 10.1186/s11671-019-3193-6

**Published:** 2019-11-28

**Authors:** Jeung Hun Park, Choong-Heui Chung

**Affiliations:** 1 Andlinger Center for Energy and the Environment, Princeton University, Princeton, NJ 08544 USA; 2 Department of Materials Science and Engineering, Hanbat National University, Daejeon, 34158 Republic of Korea

**Keywords:** Self-catalyst, Nanowires, Vapor-liquid-solid process, MOCVD, Raman spectroscopy, InP, InAs

## Abstract

We report optical phonon vibration modes in ensembles of self-catalyzed InP/InAs/InP multi core-shell one-dimensional nanostructures (nanopillars and nanocones) grown on InP(111)B substrates using liquid indium droplets as a catalyst via metal-organic chemical vapor deposition. We characterized the Raman vibration modes of InAs E_1_(TO), InAs A_1_(TO), InAs E_1_(LO), InP E_1_(TO), InP A_1_(LO), and InP E_1_(LO) from the ensemble of as-grown nanostructures. We also identified second-order Raman vibration modes, associated with InP E_1_(2TO), E_1_(LO+TO), and E_1_(2LO), in the InP/InAs/InP core-shell nanopillars and nanocones. Raman spectra of InP/InAs/InP nanopillars showed redshift and broadening of LO modes at low-frequency branches of InAs and InP. Due to the polar nature in groups III–V nanowires, we observed strong frequency splitting between InAs E_1_(TO) and InAs A_1_(LO) in InP/InAs/InP nanocones. The Raman resonance intensities of InP and InAs LO modes are found to be changed linearly with an excitation power. By tilting the substrate relative to the incoming laser beam, we observed strong suppression of low-frequency branch of InP and InAs LO phonon vibrations from InP/InAs/InP nanocones. The integrated intensity ratio of InP E_1_(TO)/E_1_(LO) for both nanostructures is almost constant at 0-degree tilt, but the ratio of the nanocones is dramatically increased at 30-degree tilt. Our results suggest that Raman spectroscopy characterization with a simple substrate tilting method can provide new insights into non-destructive characterization of the shape, structure, and composition of the as-grown nanostructures for the wafer-scale growth and integration processing of groups III–V semiconducting hetero-nanostructures into nanoelectronics and photonics applications.

## Background

Semiconducting heterostructure nanowires have received considerable attention over the past decade [1]. A variety of material combinations have been synthesized both in core-shell [2–5] and superlattice [6–8] and alloy nanowires [9, 10]. InP-InAs nanowire [11–13] is one of such combinations with potential applications in light-emitting diodes [14], single-photon source [15], photodetectors [16], and heterojunction transistors [17] due to its band gap tunability, high carrier mobility, and large breakdown field [18, 19]. The performance of any of these devices depends on the optical and electronic properties of nanoscale semiconductors, which in turn vary critically with the crystallinity, morphology, and composition of the nanowires [20, 21]. Among a suite of available characterization tools, Raman spectroscopy is a non-destructive technique that can provide insights into the effects of shape, structure, and composition of semiconductor structures (i.e., thin films [22], nanowires [23], and quantum dots [24]) on physical properties (i.e., phonon confinement and surface optical phonon modes [25, 26]). Polarization-dependent Raman scattering measurements on single semiconducting nanowires revealed that highly anisotropic shapes of nanowires have angular dependences of Raman active modes and scattered intensities (i.e., Si [27], GaAs [28], InAs [29, 30], GaP [31, 32], ZnO [33], GaN [34]). Recent advances of Raman spectroscopy technique further achieved the single-molecule level sensitivity of Raman signals through the exploitation of near-field surface resonances [35, 36] using engineered substrates with roughened metal-coated two-dimensional surface (i.e., metal nanoparticle–decorated substrate [37]) or in the form of zero-dimensional metal particles (i.e., core-shell nanoparticles [35]). By tuning the shell thickness, core size, and materials of core-shell nanoparticles, this technique can find extensive applications in chemical sensing and imaging, thermal therapy, nanophotonics, plasmon-induced photocatalysis, plasmon-enhanced signal amplifications, and fluorescence [35, 36, 38, 39]. However, Raman spectroscopic characterization of the self-catalyzed growth of one-dimensional hetero-nanostructures has not been extensively studied yet. The variations in analytical parameters (i.e., peak positions, line width, and intensities) of obtained Raman spectra can explain the scientific details of the composition, chemical environment, and crystalline/amorphous in nanostructured materials [40]. Non-destructive optical characterization on as-grown samples would provide useful information to understand their novel chemical and physical properties of unique one-dimensional hetero-nanostructures.

In this Letter, we present the results from Raman spectroscopic studies of self-catalyzed InP/InAs/InP multi core-shell nanopillars and nanocones with their strong dependencies of the Raman vibration modes and intensities on the morphology, crystal structure, and scattering geometry of the one-dimensional nanostructures.

## Methods

**Fig. 1:**
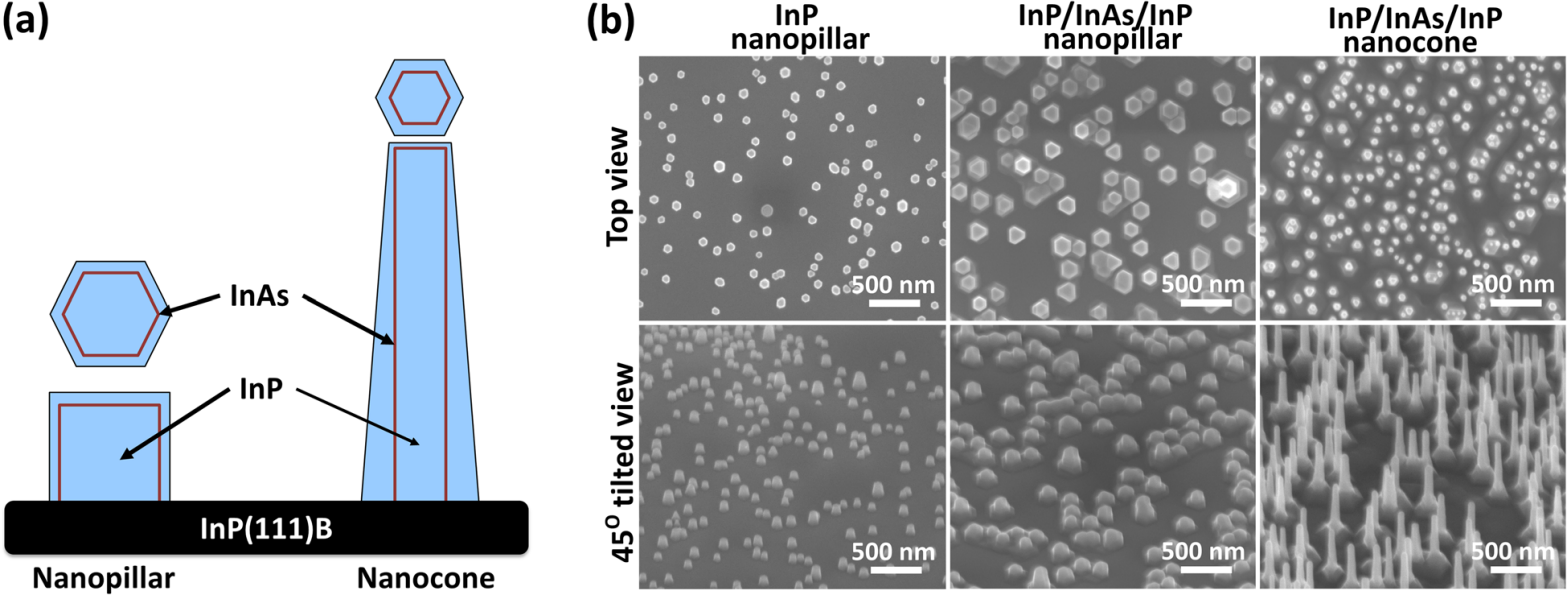
Growth morphology of InP/InAs nanostructures. **a** Schematic layout of InP/In(As,P) multi core-shell nanopillar and nanocone. **b** SEM images of top view (upper row) and 45-degree tilted view (lower row) of InP nanopillars, InP/InAs/InP nanopillars, and InP/InAs/InP nanocones grown on (111)B oriented InP single crystal wafer

One-dimensional nanostructures (nanopillars and nanocones) were grown via self-catalyzed vapor-liquid-solid process on InP(111)B substrate by a Veeco D125 MOVPE reactor using trimethylindium (TMIn), tertiarybutylphosphine (TBP), and tertiarybutylarsine (TBA) as precursors [13, 23, 41]. Nanopillars and nanocones were grown at substrate temperatures of ~ 350 °C and ~ 400 °C, respectively. In both cases, indium droplets were deposited in situ by feeding 5.06 × 10^−5^ mol/min of TMIn for 12 s. Then TMIn and TBP were introduced into the reactor at flow rates of 3.74 × 10^−6^ and 3.37 × 10^−4^ mol/min (V/III ratio = 90), respectively, to grow the InP nanostructure. After a 540-s deposition, the reactor was purged with H_2_ for 10 s and then with TBA for 180 s while the temperature was ramped up to 420 °C. Following the temperature ramp, the InAs shell was deposited onto the InP nanostructure by flowing TBA at 9.82 × 10^−3^ mol/min with a TMIn flow of 8.18 × 10^−5^ mol/min (V/III ratio = 120). The InAs growth time was 10 s. The reactor was purged with H_2_ for 10 s and with TBP for 60 s, and an InP cap layer was deposited by feeding 3.73 × 10^−6^ mol/min of TMIn and 3.37 × 10^−3^ mol/min of TBP (V/III ratio = 90) for 60 s. Finally, the sample was cooled while flowing H_2_ gas and the reactor maintained at 60 Torr. Pure InP nanopillar and nanoisland samples are prepared using the same procedure as above, except that the final InAs shell deposition step was omitted (see Fig. 1a and Additional file 1: Figure S1).

The as-grown samples analyzed in this experiment comprised vertically oriented nanostructures, grown on InP(111)B substrate. After deposition, we examined the morphology of as-grown nanostructures using a FEI NOVA 230 field emission SEM at an acceleration voltage of 5 kV. From the SEM images, we measured the average height and base diameter of over 30 individual nanostructures. Raman spectra of the as-grown samples, ensembles of InP/InAs/InP nanopillars or nanocones, were measured in backscattering geometry with confocal configuration using a Renishaw InVia Raman spectrometer. In order to avoid any Raman scope-induced physical damages on the as-grown nanostructures, a substrate tilting angle was limited up to 35 degrees. In this system, the incident laser wavelength is 514.5 nm and excitation power can be varied between 5 and 25 mW. The laser beam was focused through a microscope to a spot size of approximately 1 μm in diameter. The spectra were characterized with a resolution of 0.5 cm^−1^. All spectra were collected in air, at room temperature, and are calibrated to the reference Si peak arising from the substrate (520.1 cm^−1^). All the Raman spectra were fit with symmetric Gaussian-Lorentzian functions to extract the parameters of interest.

## Results and Discussion

Figure 1 shows typical morphologies of InP nanopillars, InP/InAs/InP nanopillars, and InP/InAs/InP nanocones grown on InP(111)B substrates. InP/InAs multi core-shell nanostructures are grown in the temperature range from 320 to 400 °C. All nanostructures grow vertically and straight in the <111>B direction with slight tapering. The pillars are low profile due to the two competitive growth modes, vapor-liquid-solid and vapor phase epitaxy, that are active at a relatively high growth temperature of 400 °C [13, 41]. The nanopillars are 150 nm in base diameter and up to 250 nm in height while the nanocones are 50 nm in base diameter and up to 2 μm in height. Detailed structural characterizations are described in [42].

**Fig. 2:**
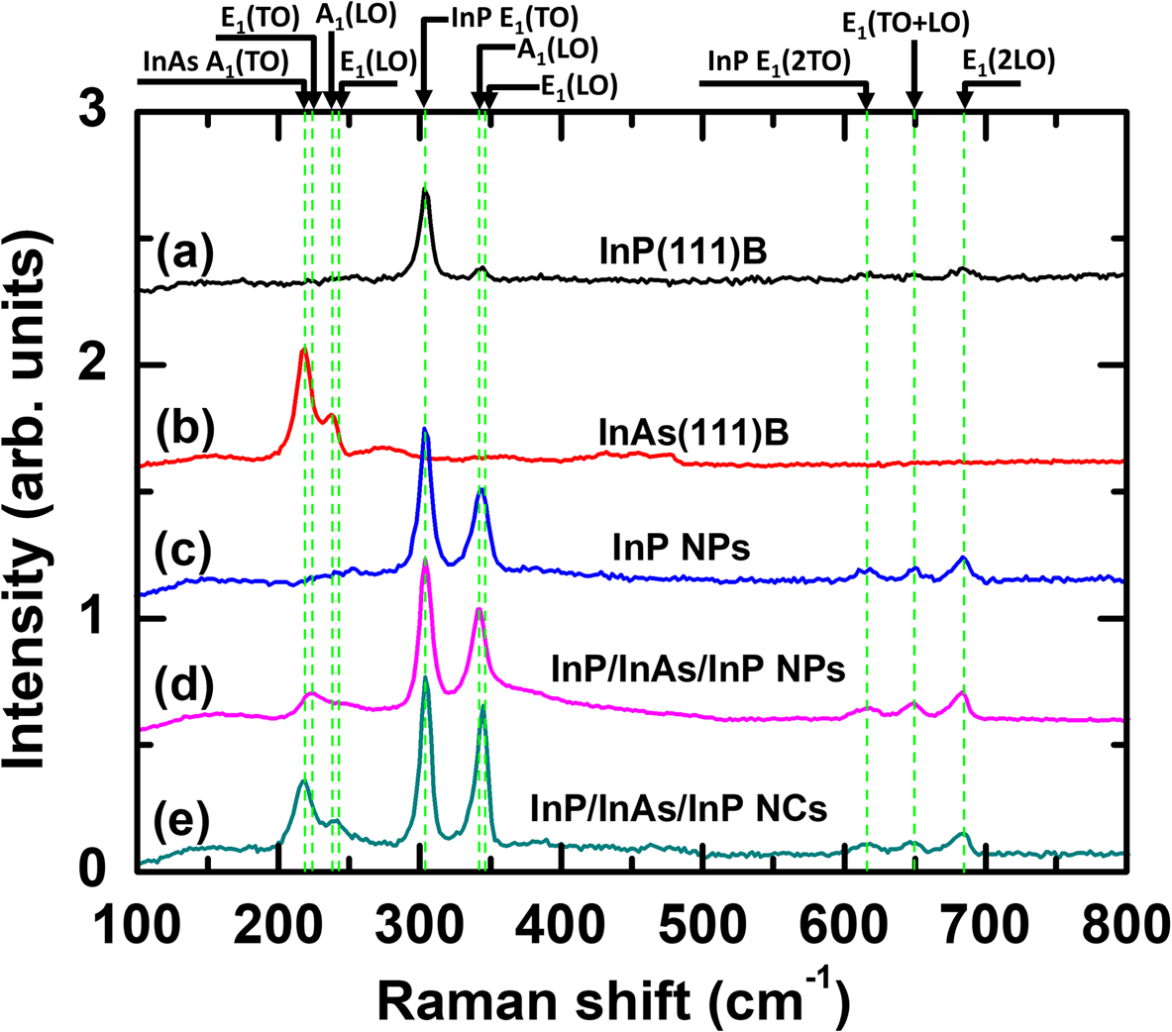
Raman spectra of (**a**) InP(111)B crystal, (**b**) InAs(111)B crystal, (**c**) InP nanopillar, (**d**) InP/InAs/InP nanopillar, and (**e**) InP/InAs/InP nanocones. The green dot lines are corresponded to InAs A_1_(TO), InAs E_1_(TO), InAs A_1_(LO), InAs E_1_(LO), InP E_1_(TO), InP A_1_(LO), InP E_1_(LO), InP E_1_(2TO), InP E_1_(TO+LO), and InP E_1_(2LO) in sequence

Figure 2 shows a series of Raman spectra obtained from InP and InP/InAs/InP nanocone and nanopillar samples with the incident laser beam oriented along the axes of the nanostructures. As the references, Raman spectra of InP thin films on InP(111)B and InAs(111)B substrates are also shown in Fig. 2. Since bulk InP crystal has a zinc blende structure (ZB) with ${T}_d^2$ space group, there is one Raman active mode of F_2_ representation that splits into transverse optical (TO) and longitudinal optical (LO) phonon modes in the polar nature of InP and InAs [43]. Phonon vibration modes within wurtzite (WZ) crystal structure of ${C}_{6v}^4$ space group are allowed in A_1_, E_1_, E_2H,_ and E_2L_. The polarity of the vibrations causes the degenerate energies of A_1_ and E_1_ modes to split into LO and TO components [44].

All the spectra from InP(111)B substrate and InP/InAs/InP nanocones exhibit two distinct peaks at 303.7 cm^−1^ and at 344.5 cm^−1^ which are assigned to be TO and LO phonon vibration modes in ZB InP bulk system, respectively. The Raman spectra for InP nanopillars in backscattering geometry mode revealed the two phonon modes at 303.8 cm^−1^ and 343.0 cm^−1^, which are consistent with the InP E_1_(TO) and InP E_1_(LO) modes for WZ structures, respectively. Interestingly, InP/InAs/InP nanopillars exhibit a noticeable enhancement and broadening of the LO band, which is not seen from InP bulk. The Raman spectra of InP/InAs/InP nanopillars at 303.8 cm^−1^ and 341.7 cm^−1^ are identified to be InP E_1_(TO) and InP A_1_(LO) modes, respectively. It is known that the LO modes are more sensitive to the Raman resonance due to the Frölich interaction [45].

The Raman peaks located at 218 cm^−1^ and 241 cm^−1^ are assigned to the first-order E_1_(TO) and E_1_(LO) modes of zinc blende InAs [46, 47] in Fig. 2. The Raman intensities of the InAs peaks in InP/InAs/InP nanostructures are lower than those of the InAs(111)B reference, indicating that both nanopillar and nanocones are either core-shell or InPAs alloy structures [13, 42]. Interestingly, the redshifts of InAs E_1_(LO) and InAs A_1_(LO) peaks compared with InAs bulk crystal with significant broadening are found in InP/InAs/InP nanopillars (see Additional file 1: Figure S2). Material size and shape (i.e., sub nanometers) can lead to a redshift and broadening of the LO Raman line [48] due to the relaxation at Г (*q* = 0) point governed by the selection rule [49]. In particular, the InAs A_1_(LO) Raman active mode confirms that WZ crystal phases are dominant in the InP/InAs/InP nanopillars [42] and our results are consistent with other reports [29, 50].

In addition to first-order Raman modes, second-order Raman modes (2TO, TO+LO, 2LO) from nanopillars and nanocones can be detected in the Raman spectra between 600 and 700 cm^−1^. The second-order harmonics correspond to singularities in the two-phonon density of states which occur when the dispersion curves are either both parallel or one is horizontal, particularly at the critical points of the Brillouin zone [51]. In contrast, these second-order phonon vibration modes are not found in the Raman spectra obtained from InP(111)B reference substrate (see Additional file 1: Figures S1 and S2). For InP/InAs/InP nanopillars, the peaks measured at 616 cm^−1^ and 649 cm^−1^ are in good agreement with the expected 2TO(Г) and TO(Г)+LO(Г) phonon modes, but the peak measured at 2LO(Г) is slightly blueshifted from the expected position. According to the phonon dispersion measurement [52], the longitudinal branch at point L is located only 4.5 cm^−1^ below the frequency we found at point Г; thus, contributions from both points presumably occur in the measured 2LO peak. For InP/InAs/InP nanocones, the peaks at 649 cm^−1^ and 684 cm^−1^ are consistent with TO(Г)+LO(Г) and 2LO(Г) phonon modes, but the peak of 2TO(Г) at 619 cm^−1^ is slightly deviated from its expected position, which may be derived from the high aspect ratio of one-dimensional nanocones [53]. All detected Raman peaks are summarized in Table 1.

**Table 1:** Raman vibration modes of InP and InAs nanostructures on InP(111)B substrate

Material	Crystal phase	Critical point	Mode	Frequency (cm^−1^)
InP	ZB	Г	E_1_(TO)	304.0
InP	WZ, ZB	X, L, Г	A_1_(LO), E_1_(LO)	339, 344.5
InP	ZB	L	E_1_(2TO)	617
InP	ZB	Г, X	E_1_(TO+LO)	650
InP	ZB	Г	E_1_(2LO)	682
InAs	ZB	Г	E_1_(TO)	218
InAs	ZB	Г	E_1_(LO)	241
InAs	WZ	L	A_1_(TO), E_1_(TO)	225.5, 226.0
InAs	WZ	L	A_1_(LO), E_1_(LO)	246.3, 246.8

**Fig. 3:**
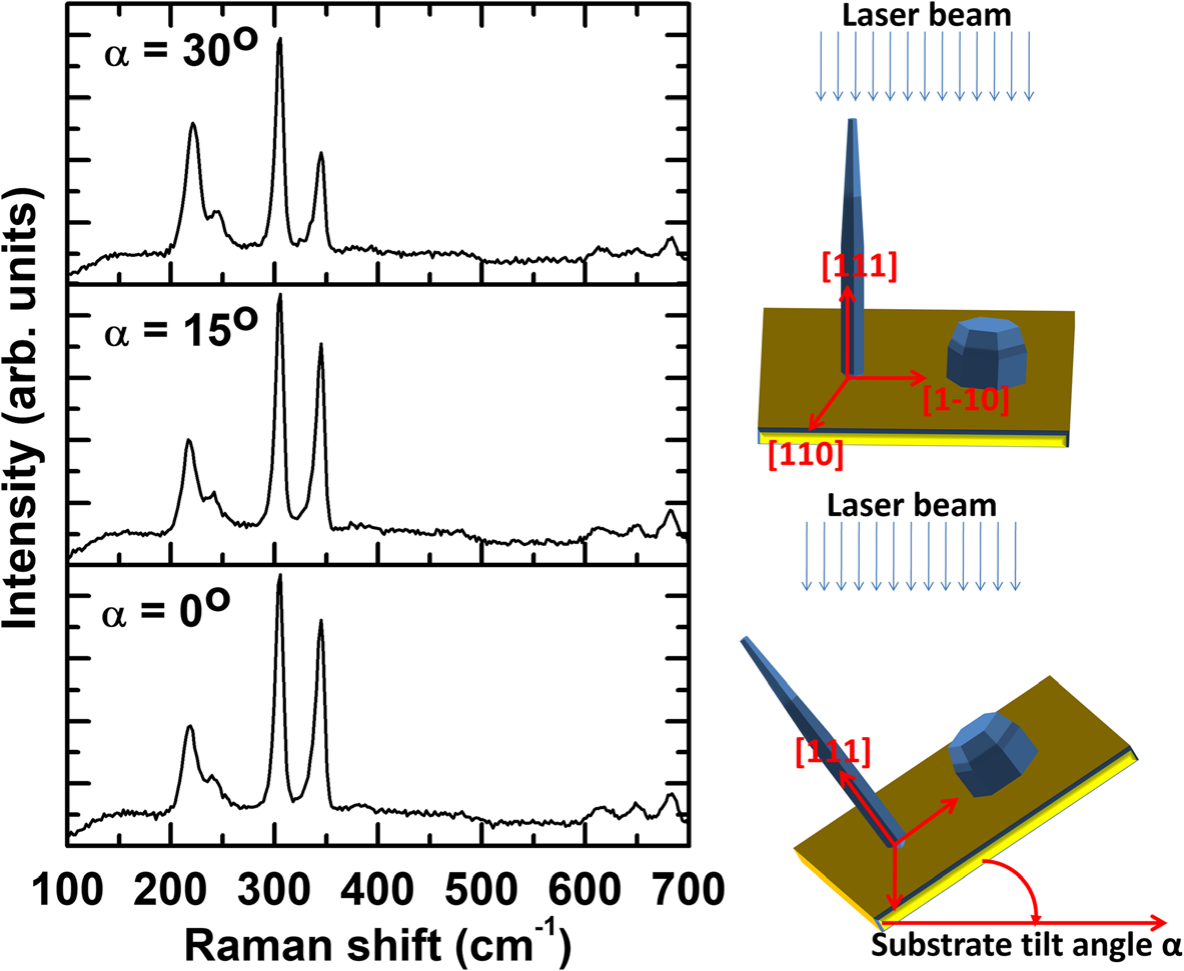
Effect of substrate tilting on Raman active modes in InP/InAs/InP nanocones

Figure 3 shows the Raman spectra of InP/InAs/InP cones, measured by varying the substrate angle from 0 to 30°. As the substrate tilt angle increased, the peak intensity corresponding to TO modes for InP and InAs is noticeably enhanced. Due to crystal symmetry between zinc blende and wurtzite [54]**,** the TO phonon is allowed in backscattering from the (110) and (111) surfaces whereas LO phonon is allowed from the (100) and (111) surfaces [28]. In Raman scattering configuration with normal incidence, the laser excitation is linearly polarized in the (111) substrate plane and the incident and backscattered vectors are orthogonal. Since nanocones and nanopillars grow along (111) surface, both TO and LO modes are allowed as shown in Fig. 2. However, due to the presence of substrate tilt, additional contribution from the (110) and (100) surface will be added into the TO and LO phonons, respectively. In our previous reports, it was determined that the nanopillars have wurtzite crystal structure oriented parallel to the [0001] axis [42] but the nanocones have zinc blende crystal structure with [111] normal to the substrate [13, 55]. The set of {1–100} planes are the side facets of the nanopillars. In fact, from the crystallographic point of view, zinc blende and wurtzite structure differ only in the stacking periodicity of the InP (or InAs) bilayers in which a bilayer consists of two stacked In and P (or As) layers; the stacking order is ABCABC for zinc blende and ABAB for wurtzite structure. Zinc blende (111) plane is parallel to wurtzite (0001)–oriented planes. As the unit cell of wurtzite structure along [0001] axis is double with respect to the zinc blende along the [111], the wurtzite phonon dispersion can be approximately by folding that of the zinc blende structure along the [111] direction [28]. Both nanocones and nanopillars in our growth experiments have hexagonal cross section with (110) sidewall facets. The reflections from (110) sidewall facets are contributed to TO mode enhancements for both InP and InAs spectra, and thus, LO mode is relatively suppressed.

**Fig. 4:**
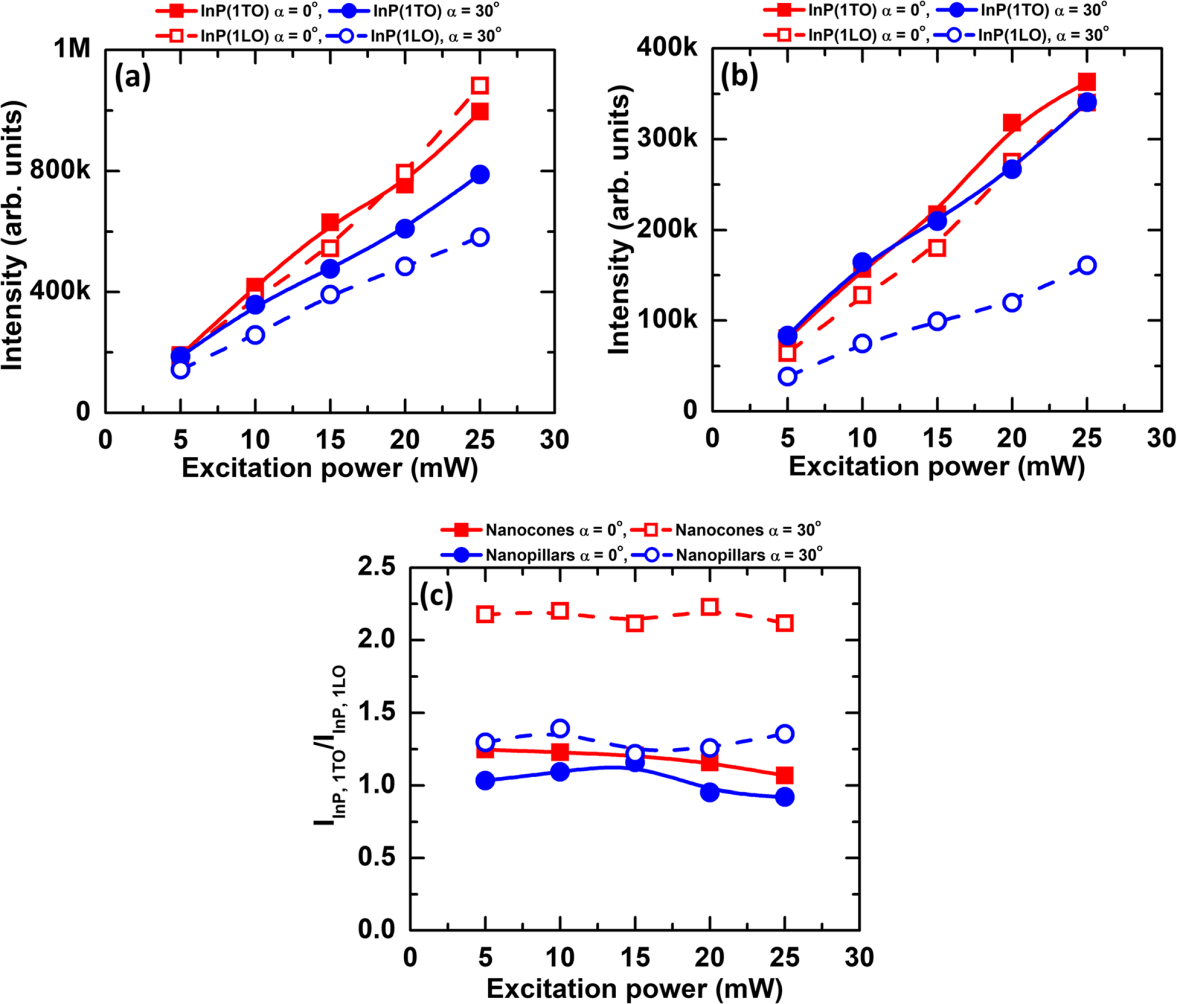
An excitation power dependence on Raman spectra of InP 1TO and InP 1LO peaks for different substrate tilts. **a** InP/InAs/InP nanopillars. **b** InP/InAs/InP nanocones. **c** Integrated intensity ratio of InP 1TO over InP 1LO excitations

Figure 4 shows an excitation power dependence of InP TO and LO peaks on Raman spectra for different substrate tilts and their relative intensity ratios I(LO, InP)/I(TO, InP). For nanopillars, a redshift (2–3 cm^−1^) of the E_1_(TO, InAs), A_1_(LO, InAs) with the broadening effect are found when the laser intensity was increased from 5 to 25 mW (see Additional file 1: Figure S2a-b). For nanocones, no substantial redshift and broadening effect are identified (see Additional file 1: Figure S2c-d). The laser heating induced Raman redshift of nanopillars was much less significant under our measurement conditions. As can be seen in Fig. 4a, b, strong Raman resonance from InP TO and LO can be found from the nanopillars due to the larger effective scattering cross section (or base diameter) of the nanopillars than that of the nanocones, respective to the incident laser beam. All of the integrated Raman intensities linearly increase with respect to the excitation power that confirms no laser heating effect under this experimental condition. By the substrate tilt, TO reflection from both nanocones and nanopillars overwhelms LO reflection (see also in Fig. 3 and Additional file 1: Figure S2). Figure 4c shows the relative integrated intensity ratio of I(TO, InP) over I(LO, InP) as a function of the excitation power. At 0-degree tilt, the integrated intensity ratio shows similar values for both nanocones and nanopillars. At 30-degree tilt, however, the ratio of nanocones (~ 2.3) becomes dramatically enhanced compared with the nanopillars (~ 1.3). Substrate tilt and excitation power dependence on Raman resonance behavior can be explained by nanowire orientation–induced cross section changes between photons and lattices [49]. The intensity ratio is strongly affected by the crystal orientation, geometry of measurement, and the surface electric field of nanowires [49, 56]. We suggest that Raman spectroscopic characterization combined with a simple substrate tilting method can be used to identify the growth morphology, crystal structure, and composition of as-grown groups III–V semiconducting hetero-nanostructures with the resolution of few nm–thick coating of InAs shell onto InP matrix.

## Conclusion

We have presented the experimental results of Raman spectroscopy performed on one-dimensional self-catalyzed InP/InAs/InP multi core-shell nanopillars and nanocones on InP(111)B substrates. The measurements are performed by varying laser power and substrate tilt angle under the fixed backscattered geometry of the Raman spectroscopy system. The InP/InAs/InP multi core-shell nanostructures exhibited the Raman resonance peaks of InAs E_1_(TO), InAs A_1_(TO), InAs E_1_(LO), InP E_1_(TO), InP A_1_(LO), and InP E_1_(LO). Contrary to the reference single-crystal InAs(111)B and InP(111)B substrates, the InP/InAs/InP nanostructure bundles revealed the unique 2nd harmonic Raman interaction modes: InP E_1_(2TO), InP E_1_(LO+TO), InP E_1_(2LO). The InP and InP/InAs/InP nanopillars showed the redshift and broadening of LO modes. Strong splitting between InAs E_1_(TO) and InAs A_1_(LO) are observed in InP/InAs/InP nanocones. We also found that the intensities of LO and TO modes are dependent linearly on an excitation power and the changes in the integrated intensity ratio of TO over LO modes are almost constant. By tilting a substrate, however, we observed a strong suppression at the low-frequency branches of the InAs LO and InP LO phonon vibrations from the InP/InAs/InP nanocone bundles, where the intensity ratio of InP TO/LO for nanopillars and nanocones is approximately 1.3 and 2.3, respectively. Our work provides new insight into the non-destructive characterization of groups III–V semiconducting hetero-nanostructures with a simple substrate tilting method.

## Supplementary information

**Additional file 1: Figure S1.** InP(111)B “nanoisland” reference substrate, treated with high temperature deposition of InP nanostructure, exhibiting preferred lateral growth to vertical growth. **Figure S2.** Effect of excitation power on Raman spectra of InP/InAs/InP nanopillar and nanocone for two substrate tilting angles (0 and 30 degrees).

## Data Availability

All data generated or analyzed during this study are included in this published article and its supplementary information files.

## Abbreviations

As: Arsine
EDS: Energy-dispersive spectroscopy
In: indium
LO: Longitudinal optical phonon
MOCVD: Metal-organic chemical vapor deposition
P: Phosphine
SEM: Scanning electron microscope
TBA: Tertiarybutylarsine
TBP: Tertiarybutylphosphine
TMIn: Trimethylindium
TO: Transverse optical phonon
WZ: Wurzite crystal structure
ZB: Zinc blende crystal structure
