# Supplementary material for: Raman Spectroscopic Characterizations of Self-Catalyzed InP/InAs/InP One-Dimensional Nanostructures on InP(111)B Substrate using a Simple Substrate-Tilting Method
Source: Nanoscale Res Lett. 2019 Nov 28;14:355. doi: 10.1186/s11671-019-3193-6 (PMC6883012; doi:10.1186/s11671-019-3193-6)
Supplement: Supplementary file 1 — Additional file 1: Figure S1. InP(111)B “nanoisland” reference substrate, treated with high temperature deposition of InP nanostructure, exhibiting preferred lateral growth to vertical growth. Figure S2. Effect of excitation power on Raman spectra of InP/InAs/InP nanopillar and nanocone for two substrate tilting angles (0 and 30 degrees). [file 11671_2019_3193_MOESM1_ESM.pdf]

Electronic Supplementary Materials for

Raman Spectroscopic Characterizations of Self-Catalyzed InP/InAs/InP One-Dimensional Nanostructures on InP(111)B Substrate using a Simple Substrate-Tilting Method

Jeung Hun Park[1,*] and Choong-Heui Chung[2,*]

[1]Andlinger Center for Energy and the Environment, Princeton University, Princeton, New Jersey 08544, United States
[2]Department of Materials Science and Engineering, Hanbat National University, Daejeon 34158, Republic of Korea.

*Correspondence: jeungp@princeton.edu (Dr. J. H. Park) and choong@hanbat.ac.kr (Prof. C.-H. Chung)

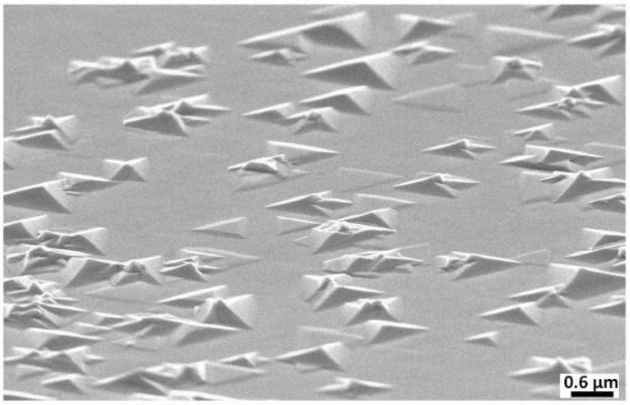

Fig. S1 InP(111)B "nanoisland" reference substrate, treated with high temperature deposition of InP nanostructure, exhibiting preferred lateral growth to vertical growth.

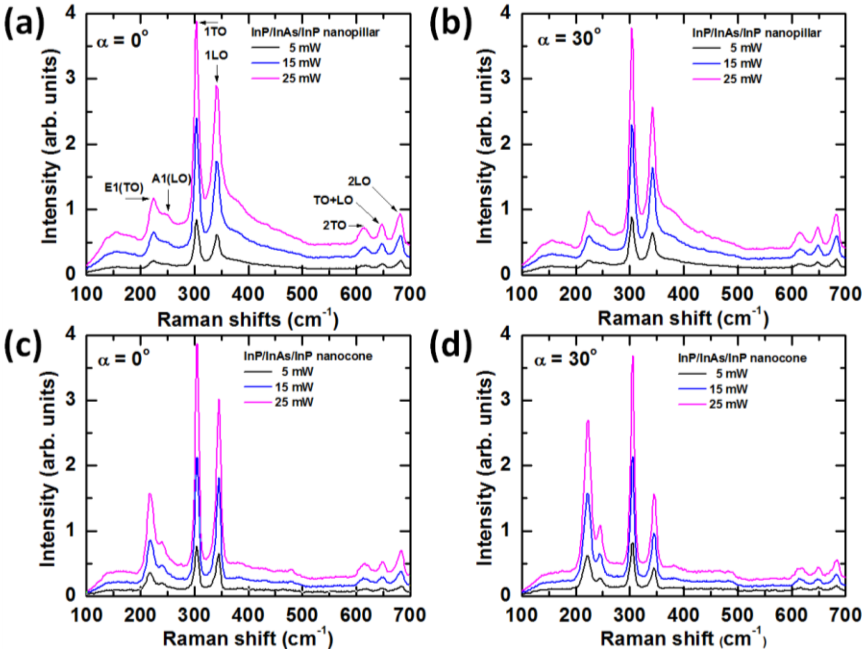

Fig. S2 Effect of excitation power on Raman spectra of (a) InP/InAs/InP nanopillar, (b) the nanopillar with 30 degree tilt, (c) InP/InAs/InP nanocone, and (d) the nanocone with 30 degree tilt.
